# High-Sensitivity C-Reactive Protein Levels in Metabolic Dysfunction-Associated Steatotic Liver Disease (MASLD), Metabolic Alcohol-Associated Liver Disease (MetALD), and Alcoholic Liver Disease (ALD) with Metabolic Dysfunction

**DOI:** 10.3390/biom14111468

**Published:** 2024-11-18

**Authors:** Seong-Uk Baek, Jin-Ha Yoon

**Affiliations:** 1 Graduate School, Yonsei University College of Medicine, Seoul 03722, Republic of Korea; 2 Department of Preventive Medicine, Yonsei University College of Medicine, Seoul 03722, Republic of Korea; 3 The Institute for Occupational Health, Yonsei University College of Medicine, Seoul 03722, Republic of Korea

**Keywords:** metabolic disease, inflammation, oxidative stress, inflammatory marker, liver disease, high-sensitivity C-reactivity protein

## Abstract

Metabolic dysfunction-associated steatotic liver disease (MASLD) is a recently introduced term for steatotic liver disease (SLD). Although the inflammatory process is central to the pathogenesis of SLD, research investigating the differences in systemic inflammation across various SLD subtypes as well as sex differences is limited. This population-based, cross-sectional study investigated the association between SLD subtypes and high-sensitivity C-reactive protein (hs-CRP) levels among Korean adults (N = 20,141; mean age: 50.8 ± 16.7 years). The participants were classified into five groups that included no SLD, MASLD, metabolic alcohol-associated liver disease (MetALD), alcoholic liver disease with metabolic dysfunction (ALD with MD), and other SLDs. The median (Q1, Q3) value of the hs-CRP level was 0.54 mg/L (0.33, 1.04). Among men, compared to levels in the no SLD group, the MASLD, MetALD, and ALD with MD groups were associated with 41.9% (95% confidence interval [CI]: 35.1–49.1%), 46.8% (95% CI: 35.0–59.6%), and 51.8% (95% CI: 30.0–77.2%) increases in hs-CRP levels, respectively. The association between SLD subtypes and hs-CRP levels was stronger among women, and compared to the levels in the no SLD group, the MASLD, MetALD, and ALD with MD groups were associated with 81.5% (95% CI: 73.6–89.8%), 84.3% (95% CI: 58.1–114.8%), and 98.2% (95% CI: 38.0–184.8%) increases in hs-CRP levels, respectively. In conclusion, our findings indicate a varying profile of systemic inflammation across SLD subtypes, with more pronounced increases in hs-CRP levels in women with SLDs.

## 1. Introduction

Steatotic liver disease (SLD) is among the most prevalent liver conditions [1,2,3]. The prevalence of SLD in South Korea has increased steadily due to factors such as an aging population, shift towards Westernized dietary habits, and sedentary lifestyle [4,5,6,7], posing a substantial health burden. The concept and nomenclature of SLD have recently undergone considerable changes, shifting from nonalcoholic fatty liver disease (NAFLD) to metabolic dysfunction-associated steatotic liver disease (MASLD) [8]. While the definition of NAFLD relies on the exclusion of individuals with significant alcohol consumption, the newly introduced MASLD concept explicitly requires the presence of metabolic dysfunction (MD) [8,9]. The diagnosis of MD is established if at least one of the following five cardiometabolic risk factors (CMRFs) is present: overweight or obesity, prediabetes or diabetes mellitus, elevated blood pressure, high triglyceride level, or low high-density lipoprotein cholesterol level [8]. The concept of the MASLD is now accepted as the standard for classifying and managing SLD, introducing significant conceptual shifts in both clinical practice and research [10,11].

One of the key aspects of the newly proposed concept of SLD is that unlike the past classification that mutually excluded NAFLD and alcoholic liver disease (ALD), SLD is considered a spectrum. For example, while MASLD is defined as individuals with SLD and MD without significant alcohol consumption, those with both MD and significant alcohol intake can be categorized as having metabolic alcohol-associated liver disease (MetALD) or ALD with MD according to the amount of alcohol consumption [12,13,14]. This spectrum-based approach, ranging from MASLD to MetALD and ALD to MD, reflects a real-world situation in which alcohol consumption and MD frequently coexist in individuals with SLD. Compared to the previous classification of NAFLD, the newly introduced SLD concept has gained increasing support as a more effective tool for identifying individuals at risk of cardiovascular diseases (CVDs) and mortality [15,16,17,18].

SLD is characterized by the accumulation of triglycerides in liver that triggers a sequence of pathological changes, including alterations in adipokine secretion, lipotoxicity, and inflammation [9,19]. These changes ultimately result in metabolic dysfunction-associated steatohepatitis (MASH) and liver cirrhosis [20]. Previous studies have demonstrated that the levels of proinflammatory cytokines are elevated in patients with NAFLD or MASLD compared to those in healthy individuals. For example, inflammatory markers such as tumor necrosis factor-α (TNF-α), interleukin-6 (IL-6), interleukin-1β (IL-1β), and C-reactive protein (CRP) were elevated in individuals with NAFLD or MASLD [21,22,23]. C-reactive protein (CRP) is a well-known inflammatory marker of the liver; its secretion is primarily stimulated by IL-6 [24,25,26]. In particular, the severity of the inflammatory response in patients with SLD is significant, as elevated inflammation correlates with a greater degree of steatosis and an increased risk of fibrosis [27].

Chronic low-grade systemic inflammation is associated with CVD and mortality. For example, previous studies have consistently demonstrated that high-sensitivity CRP (hs-CRP) is a reliable biomarker for predicting the risk of CVD occurrence [28,29]. For example, hs-CRP levels of >3 mg/L indicate a high risk of CVD based on the guideline of the American Heart Association [30]. Systemic inflammation also has a significant clinical relevance in individuals with SLD. Chronic systemic inflammation is associated with the mediation of the impact of MASLD or MASH on CVD mortality and chronic kidney disease [31]. In individuals with NAFLD, elevated hs-CRP levels are associated with an increased risk of CVD [32]. Elevated hs-CRP levels also play a mediating role in the relationship between MASLD, CVD onset, and mortality [33,34].

Substantial sex differences in the pathogenesis and epidemiology of SLD have been documented. The prevalence of SLD is higher in men than in women [7,35]. Compared to women, men are susceptible to visceral obesity, the main source of free fatty acids (FFAs). In premenopausal women, estrogen promotes gluteal-femoral adipose distribution, which has a more favorable metabolic profile than that of central obesity [36,37,38,39]. However, studies based on the histological diagnoses of steatohepatitis have demonstrated that females are at a greater risk of developing steatohepatitis and fibrosis than males during the progression of SLD [40,41,42,43]. Additionally, the increase in overall mortality due to SLD is more pronounced in females than in males [44]. However, the precise mechanisms underlying the observed sex differences in the prognosis and progression of SLD remain unclear. This study aimed to explore systemic inflammation in MASLD, MetALD, and ALD with MD with a focus on sex differences using hs-CRP as a biomarker. Particularly, we aimed to explore this association based on a large-scale, population based sample of Korean adults.

## 2. Materials and Methods

### 2.1. Study Design and Participations

This study included participants from the Korea National Health and Nutrition Examination Survey (KNHANES) conducted between 2015 and 2018, during which their hs-CRP levels were measured. KNHANES is a nationwide annual cross-sectional survey aimed at assessing the health status of the general Korean population [45]. It is conducted by the Korea Disease Control and Prevention Agency [46]. A nationally representative sample of the Korean population was selected using multistage clustered probability sampling, and household surveys were conducted by health professionals employed by the Korea Disease Control and Prevention Agency. Initially, 25,334 adults aged 18 years were included in the KNHANES database. We excluded pregnant women (*n* = 1261), individuals infected with HBV or HCV (*n* = 963), and those with a history of liver cancer (*n* = 40). Additionally, participants with hs-CRP levels greater than 10 were excluded, as this indicated an acute inflammatory condition (*n* = 1369). After eliminating observations with missing data (*n* = 1560), 20,141 adults were included in this study. All study participants provided written informed consent to participate in the Korea Disease Control and Prevention Agency before undergoing the health examinations. This study was a secondary data analysis based on the KNHANES datasets, and the study protocol was reviewed and approved by the Institutional Review Board of Severance Hospital (IRB approval No. 4–2024–0953). Raw data from KNHANES are available at https://knhanes.kdca.go.kr/ (accessed on 23 August 2024).

### 2.2. Definition of SLD and Its Subtypes

SLD subtypes were classified according to the hepatic steatosis index (HSI), presence of CMRFs, and alcohol consumption as outlined in Table 1 [11,12]. HSI was calculated based on the participants’ alanine aminotransferase (ALT)/aspartate aminotransferase (AST) ratio, diabetes status, and sex. The HSI was developed using data from the Korean population [47]. Hepatic steatosis was defined as HSI > 31 based on a recent validation study that reported a sensitivity of 88% and specificity of 72% for identifying SLD at this cutoff [48]. For the sensitivity analysis, we used a more stringent cutoff of HSI > 36 that has been previously used to classify SLD, offering lower sensitivity but higher specificity [49,50,51].

CMRFs that were used to determine the presence of MD included overweight status or obesity, prediabetes or diabetes, elevated blood pressure, high triglyceride levels, and low high-density lipoprotein cholesterol levels (Table 1). Height, weight, and waist circumference were objectively measured by health professionals following a standardized protocol to the nearest 0.1 kg and 0.1 cm. Height was measured using a SECA 274 stadiometer (SECA, Hamburg, Germany), and weight was measured using a GL-6000-20 scale (G-tech, Uijeongbu-si, Republic of Korea). Waist circumference was measured using a SECA 200 tape measure (SECA, Hamburg, Germany). Blood pressure was measured using a mercury sphygmomanometer (Baumanometer Wall Unit 33, 0850; Baum Co., Inc., Copiague, NY, USA). Serum levels of fasting glucose, glycated hemoglobin A1c, triglycerides, and low high-density lipoprotein cholesterol were measured from blood samples. Blood samples were collected via venipuncture, immediately processed, refrigerated, and transported to a central laboratory for analysis. Blood samples were collected in 3-mL EDTA-coated tubes, and serum samples were stored at temperatures ranging from 2 to 8 degrees Celsius. All laboratory analyses were performed within 24 h of sample collection. The enzyme assay was performed using the Hitachi Automatic Analyzer 7600-210 (Hitachi, Tokyo, Japan). All participants fasted for at least 8 h before blood sample collection.

Individuals were classified into the following five groups according to their HSI value, the presence of CMRFs, and alcohol consumption: (i) no SLD: HSI ≤ 31; (ii) MASLD: HSI > 31, ≥1 CMRF, and alcohol consumption < 20 g/day (women) or <30 g/day (men); (iii) MetALD: HSI > 31, ≥1 CMRF, and alcohol consumption 20–50 g/day (women) or 30–60 g/day (men); (iv) ALD with MD: HSI > 31, ≥1 CMRF, and alcohol consumption > 50 g/day (women) or >60 g/day (men); (v) other SLDs: HSI > 31 and does not meet criteria for (ii)–(iv). Alcohol consumption was determined based on the self-reported frequency and the average number of drinks consumed per sitting.

### 2.3. Hs-CRP

Hs-CRP was collected and analyzed according to the standard blood sample collection and analysis procedures described above. Serum hs-CRP levels were assessed by immunoturbidimetry using a Cobas analyzer (Roche, Hamburg, Germany) with Roche Cardiac C-Reactive Protein High-Sensitive Reagent (Roche, Hamburg, Germany). The lower detection limit (LOD) was 0.15 mg/L, and for values below this limit, half of the LOD value was used. As KNHANES was designed to conduct a large-scale health screening, it did not include the specific biomarkers, IL-1β, IL-6, and TNF-α, necessary for an in-depth investigation of systemic inflammatory processes. Considering that hs-CRP is a widely used clinical biomarker with well-documented associations with various diseases [30,52], it was employed to evaluate systemic inflammation in this study.

### 2.4. Statistical Approach

We first explored the distribution of hs-CRP values according to SLD subtypes using a box plot and the Wilcoxon rank-sum test. Prior to regression analysis, the normality of the distribution of hs-CRP values among the samples was examined using a histogram and Q-Q plot. To achieve a normal distribution, hs-CRP values were naturally log-transformed and included as dependent variables in the regression analyses. To explore the association between SLD subtypes and hs-CRP levels, we employed log-linear regression models to estimate percentage (%) changes and 95% confidence intervals (CIs) compared to levels in the reference group (no SLD). In Model 1, we examined the association between SLD subtypes and hs-CRP levels in the overall sample. Covariates included sex, age, income level, education level, smoking status, and physical activity level. Age was included as a continuous variable. Participants’ income level was categorized as lowest, low, high, or highest based on the quartile values of household income for each year. Educational attainment was grouped into middle school or lower, high school, or college or above. Current smoking status was classified as yes or no. Physical activity was classified as yes or no based on whether study participants engaged in ≥150 min of regular physical activity, as determined by the Korean Global Physical Activity Questionnaire [53]. Sex differences in the association of SLD subtypes with hs-CRP values were determined by including interaction terms (SLD subtype × sex) in Model 1. Subsequently, we conducted a sex-stratified analysis to explore how the association of SLD subtypes with hs-CRP manifested differently in men and women (Model 2). For additional analysis, we explored the association between SLD categories and AST and ALT on log-linear regression models. Moreover, we conducted logistic regression models to examine the association between sociodemographic features and the presence of SLD, defined as HSI > 31. Statistical significance was set at *p* < 0.05. Analyses and visualizations were performed using R (version 4.4.1). Survey weights were adjusted for all regression models to reflect the KNHANES sampling procedure.

## 3. Results

**Figure 1:**
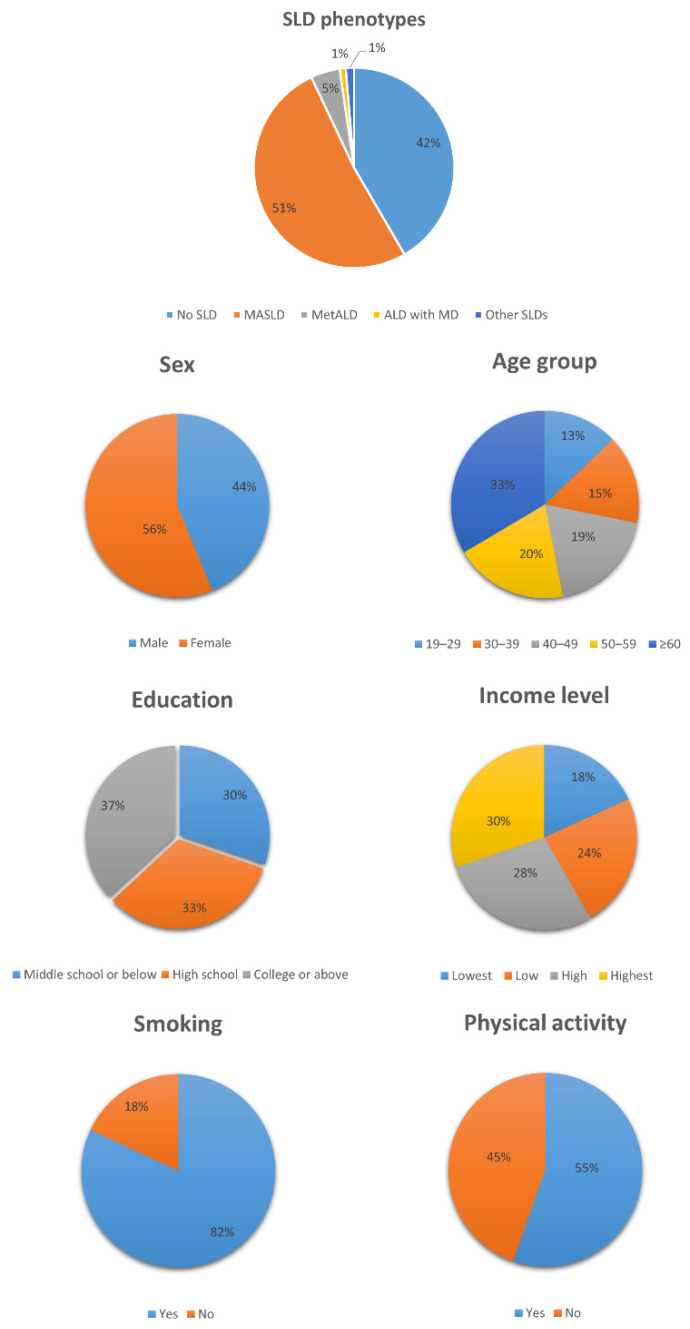
Distribution of characteristics among the study sample (SLD, steatotic liver disease; MASLD, metabolic dysfunction-associated steatotic liver disease; MetALD, metabolic alcohol-associated liver disease; ALD with MD, alcoholic liver disease with metabolic dysfunction).

Table 2 indicates the characteristics of the study sample according to the SLD categories. The study sample consisted of 8813 men (43.8%) and 11,328 women (56.2%) with a mean age of 50.8 years. The prevalence rates of no SLD, MASLD, MetALD, ALD with MD, and other SLDs were 41.6%, 51.4%, 4.7%, 1.0%, and 1.3%, respectively. Compared to those without SLD, the proportions of men, older individuals, and individuals with lower educational and income levels were higher among those with MASLD. Additionally, the median values of AST were 19.0, 21.0, 24.0, 24.0, and 19.0 for no SLD, MASLD, MetALD, ALD with MD, and other SLDs, respectively. Similarly, the median values of ALT were 13.0, 21.0, 25.0, 25.0, and 21.0 for no SLD, MASLD, MetALD, ALD with MD, and other SLDs, respectively. Figure 1 also shows the distribution of characteristics among the study sample.

**Figure 2:**
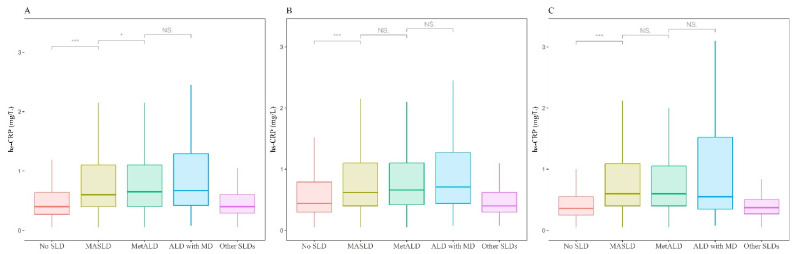
Distribution of hs-CRP (high-sensitivity C-reactive protein) values according to SLD subtypes (SLD, steatotic liver disease). (**A**) Median (Q1, Q3) values of hs-CRP in the overall sample, (**B**) median (Q1, Q3) values of hs-CRP in the male sample, and (**C**) median (Q1, Q3) values of hs-CRP in the female sample. Wilcoxon rank sum test was employed (* *p* < 0.05, *** *p* < 0.001). Abbreviation: MASLD, metabolic dysfunction-associated steatotic liver disease; MetALD, metabolic alcohol-associated liver disease; ALD with MD, alcoholic liver disease with metabolic dysfunction; NS: non-significant differences.

Figure 2 presents the distribution of hs-CRP levels according to the SLD categories. The median values of hs-CRP among the overall sample were 0.4, 0.7, 0.7, 0.7, and 0.4 for no SLD, MASLD, MetALD, ALD with MD, and other SLDs, respectively. The median values of hs-CRP among the male sample were 0.5, 0.7, 0.7, 0.7, and 0.4 for no SLD, MASLD, MetALD, ALD with MD, and other SLDs, respectively. The median values of hs-CRP among the female sample were 0.4, 0.7, 0.7, 0.7, and 0.4 for no SLD, MASLD, MetALD, ALD with MD, and other SLDs, respectively.

**Figure 3:**
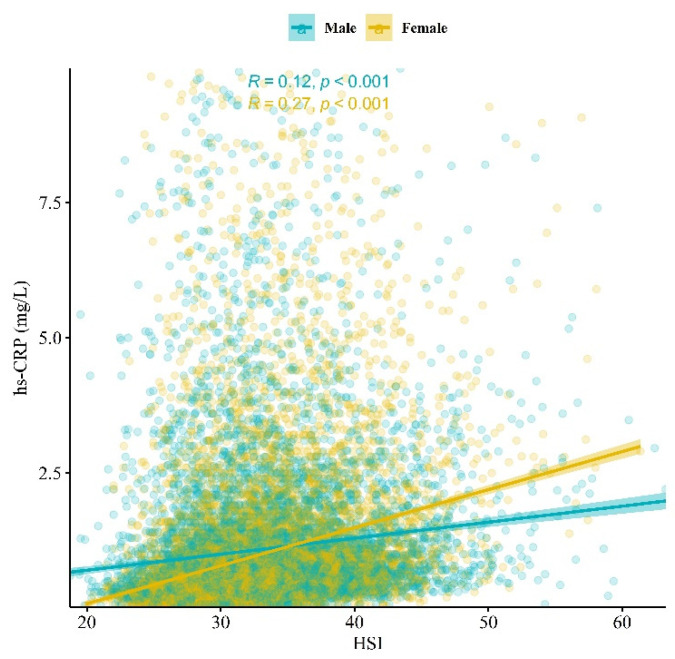
Scatter plot of hepatic steatosis index (HSI) and high-sensitivity C-reactive protein (hs-CRP) levels based on sex. Pearson’s correlation coefficients were presented.

Figure 3 presents a scatter plot of HSI and hs-CRP values among the samples. The Pearson’s coefficient between HSI and hs-CRP was 0.12 (*p* < 0.001) among men and 0.27 (*p* < 0.001) among women.

**Figure 4:**
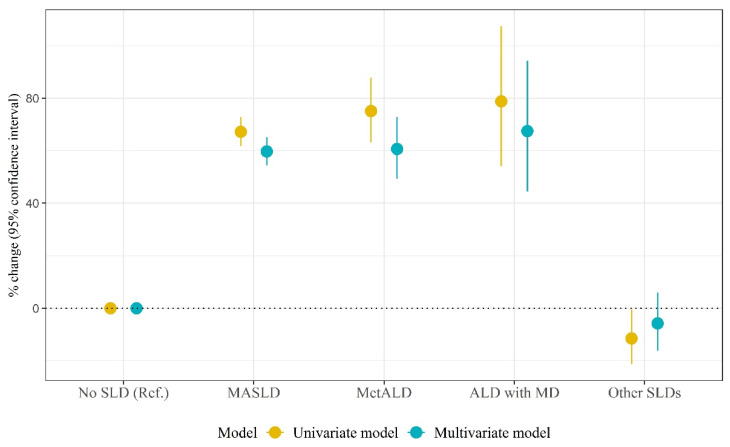
The plot depicting the association between SLD categories and hs-CRP levels among the overall sample (SLD, steatotic liver disease; MASLD, metabolic dysfunction-associated steatotic liver disease; MetALD, metabolic alcohol-associated liver disease; ALD with MD, alcoholic liver disease with metabolic dysfunction; hs-CRP; high-sensitivity C-reactive protein).

Table 3 presents the association between the SLD categories and hs-CRP levels using log-linear regression models. Compared to levels in non-SLD, MASLD, MetALD, and ALD with MD were associated with 59.7% (95% CI: 54.5%, 65.1%; *p* < 0.001), 60.6% (95% CI: 49.3%, 72.8%; *p* < 0.001), and 67.5% (95% CI: 44.4%, 94.2%; *p* < 0.001) increases in hs-CRP values, respectively (multivariate model). Table S1 presents the results of the models with interaction terms. There were significant interactions between MASLD and females (*p* < 0.001) and between MetALD and females (*p* = 0.016) for hs-CRP values. The plot depicting the association between SLD subtypes and hs-CRP among the overall sample is depicted in Figure 4.

**Figure 5:**
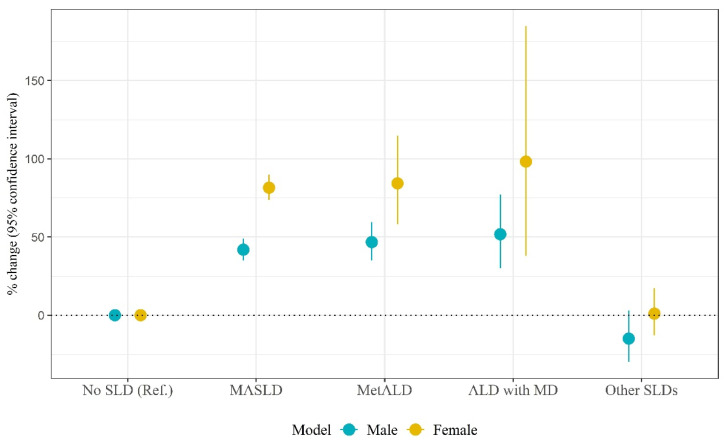
The plot depicting the association between SLD categories and hs-CRP levels among the male and female samples (SLD, steatotic liver disease; MASLD, metabolic dysfunction-associated steatotic liver disease; MetALD, metabolic alcohol-associated liver disease; ALD with MD, alcoholic liver disease with metabolic dysfunction; hs-CRP; high-sensitivity C-reactive protein).

Table 4 presents the results of sex-stratified analyses of the association between SLD categories and hs-CRP values. Among men, compared to non-SLD, MASLD, MetALD, and ALD with MD were associated with 41.9% (95% CI: 35.1%, 49.1%; *p* < 0.001), 46.8% (95% CI: 35.0%, 59.6%; *p* < 0.001), and 51.8% (95% CI: 30.0%, 77.2%; *p* < 0.001) increases in hs-CRP values, respectively. Among women, MASLD, MetALD, and ALD with MD were associated with 81.5% (95% CI: 73.6%, 89.8%; *p* < 0.001), 84.3% (95% CI: 58.1%, 114.8%; *p* < 0.001), and 98.2% (95% CI: 38.0%, 184.8%; *p* < 0.001) increases in hs-CRP values, respectively in comparison to non-SLD. The plot depicting the association between SLD subtypes and hs-CRP among the overall sample is depicted in Figure 5.

Tables S2 and S3 indicate the association between SLD categories and hs-CRP levels using an alternative cutoff (HSI > 36). Elevated hs-CRP levels were observed in MASLD, MetALD, and ALD with MD groups, with these associations being particularly pronounced among the female sample population. Tables S4–S6 show that both AST and ALT levels were significantly elevated among those with MASLD, MetALD, and ALD with MD, compared with those without SLD. Table S7 shows that men, those with older age, and those with lower educational levels, were more likely to have SLDs.

## 4. Discussion

This study found that individuals with MASLD, MetALD, and ALD with MD exhibited significantly elevated hs-CRP levels compared to those without SLD. Moreover, the association of SLD subtypes with hs-CRP levels was more pronounced in women than it was in men. Although hs-CRP levels were lower in women without SLD than they were in men, the median values and distribution of hs-CRP levels among individuals with MASLD, MetALD, and ALD with MD were similar between men and women. One of the strengths of our study is the use of a large, population-based sample (N = 20,141) for analysis. Therefore, our findings suggest the need for close monitoring and management of CVD risk in individuals with MASLD, MetALD, or ALD with MD.

In our study, the correlation between HSI and hs-CRP levels was weak, while significant associations were observed between hs-CRP and SLD subtypes, including MASLD, MetALD, and ALD with MD. HSI is a measure of hepatic fat accumulation and may not, in itself, be closely associated with steatohepatitis or inflammatory status. In contrast, the classification criteria for MASLD, MetALD, and ALD with MD offer a more comprehensive assessment of metabolic abnormalities beyond hepatic steatosis, which may account for the stronger association with hs-CRP [11,12]. For example, subcomponents of the CMRF included in the MASLD classification—such as overweight or obesity, prediabetes or diabetes, and elevated blood pressure—are closely associated with systemic inflammation and hs-CRP, as indicated by previous meta-analytic reviews [54,55,56]. Therefore, SLD subtypes, including MASLD, can show stronger correlations with hs-CRP than HSI due to their inclusion of a more complex range of metabolic abnormalities.

Our findings are in line with those of prior studies that identified elevated levels of systemic inflammatory biomarkers in patients with MASLD. For example, compared to those without MASLD, those with MASLD possess elevated levels of hs-CRP, ferritin, or a systemic immune-inflammatory index [57,58,59]. These findings accord with earlier studies indicating a positive relationship between NAFLD and hs-CRP levels [60,61]. Therefore, this study supports previous findings that systemic inflammation is a key factor in the pathogenesis of SLD subtypes associated with MD, including MASLD, MetALD, and ALD with MD [20]. Additionally, previous studies have shown that male sex, older age, and lower education levels are associated with an increased risk of SLD, which aligns with the findings of our study [6,14].

The pathogenesis of MASLD is often explained by the “multiple-hit” hypothesis, in which factors such as lipotoxicity, insulin resistance, nutritional intake, gut microbiota, and genetic factors are involved in the induction of MASLD [62,63]. Studies have suggested that systemic inflammation is involved in various mechanisms during this process [20,26]. The inflammatory pathways, including JNK/AP-1 and IKK/NF-κB signaling, are activated in the process of MASLD induced by various factors including adipose tissue dysfunction, lipotoxicity, and endotoxins driven by the gut [64]. Subsequently, elevated CRP levels are involved in various mechanisms of the multiple-hit pathogenesis of MASLD, including leptin signaling, insulin signaling, and mitochondrial dysfunction, leading to the occurence and progression of MASLD [20,26]. The activation of proinflammatory markers plays a key role not only in the progression to MASH and liver fibrosis [20] but also in extrahepatic manifestations such as the onset of CVD events or mortality [31].

An intriguing finding of our study was the sex differences in the association of MASLD with hs-CRP levels. While the prevalence of MASLD, MetALD, and ALD with MD was higher among men than it was in women, the increase in hs-CRP levels compared to that of the control group without SLD was greater among women than it was among men. This is consistent with the findings of prior studies indicating that the association of hs-CRP with SLD is more pronounced among women than it is among men [65,66,67]. Additionally, women with SLD are more likely to develop steatohepatitis, advanced liver fibrosis, and mortality than are their male counterparts [40,41,42,43,44]. Although the exact mechanisms are not fully understood, one potential explanation may lie in sex differences in fat distribution. For example, compared to men, women exhibit significant differences in fat distribution, with males exhibiting a higher proportion of visceral fat and females exhibiting more subcutaneous fat [68]. Previous studies have demonstrated that visceral adipose tissue, rather than subcutaneous adipose tissue, is strongly linked to proinflammatory cytokine secretion and adverse metabolic profiles, and this explains the more favorable metabolic status and lower inflammatory status in healthy women [69]. However, in women with MASLD where visceral fat accumulation is significantly elevated, the comparative disparity in fat distribution between women with and without MASLD may be more pronounced than it is in men [70,71,72]. Furthermore, the influence of estrogen may also contribute to these sex-based disparities. Premenopausal women, exhibiting a lower prevalence of SLD, tend to have lower hs-CRP levels due to the anti-inflammatory effects of estrogen [65,66,67]. Consequently, as indicated in our study, women without SLD exhibit lower hs-CRP levels compared to men without SLD. However, postmenopausal women, who are at a higher risk for MASLD, MetALD, and ALD with metabolic dysfunction, experience a loss of estrogen’s protective effects, which contributes to elevated hs-CRP levels among women with SLDs [73,74]. This may explain the greater disparity in hs-CRP levels between women with and without SLD, compared to men. However, due to the lack of body composition data, we were unable to precisely explain the mechanisms underlying our findings.

This study possesses some limitations. First, due to the cross-sectional study design, we could not determine the temporal relationship between SLDs and hs-CRP levels that prevented us from establishing a causal link. Second, as the primary purpose of KNHANES was to screen the overall health status of a large-scale sample, it did not include measurements for an in-depth analysis of systemic inflammation, such as IL-1β, IL-6, and TNF-α, or for liver function, such as blood levels of alkaline phosphatase and gammqa-glutamyl transferase. Additionally, our investigation focused solely on hs-CRP levels, which limited our ability to assess a broader spectrum of systemic inflammation. A study has shown that in cases where active immune-mediated inflammatory diseases coexist with liver problems, CRP may not serve as a reliable inflammatory marker [8]. Nevertheless, past research has indicated that hs-CRP efficiently reflects the severity of inflammation in individuals with SLD [9,10,11,12]. Moreover, several previous studies, particularly large-scale investigations, have used hs-CRP alone to examine differences in inflammatory response among those with and without SLD [9,11,13,14]. However, combining multiple biomarkers may allow for a more accurate identification of systemic inflammation compared to relying solely on hs-CRP. Therefore, future research should include a wider range of inflammatory markers, such as IL-1β, IL-6, and TNF-α, to provide a more comprehensive understanding of the inflammatory processes associated with SLD subtypes. Third, we relied on HSI, a non-invasive measure, to determine hepatic steatosis. However, compared to imaging modalities or biopsies, the utilization of serum biomarkers is prone to misclassification errors [75]. Although the Fatty Liver Index (FLI) is one of the most widely used noninvasive biomarkers [76], we were unable to calculate it due to the lack of data regarding serum gamma-glutamyl transferase levels. Nevertheless, previous studies have widely used HSI to classify MASLD, particularly in large-scale community-based samples, with reasonable accuracy [47,48,51,77]. Fourth, several important factors such as genetic predisposition and the use of hepatotoxic medications that could exert a substantial impact on both hs-CRP and SLD were not considered due to a lack of information. Nevertheless, a significant strength of this study is its large-scale analysis based on a nationally representative sample that enhances the generalizability of the findings.

## 5. Conclusions

Using a nationwide sample of 20,141 Korean adults, this study demonstrated that individuals with MASLD, MetALD, or ALD with MD exhibited higher hs-CRP levels (indicating systemic inflammation) than did those without SLD. Additionally, the increase in hs-CRP levels in patients with MASLD, MetALD, and ALD with MD was more pronounced in women than it was in men.

## Figures and Tables

**Table 1:** Definition of SLD and its subtypes.

	Operationalization
Hepatic steatosis	Hepatic steatosis index (HSI) > 31
Cardiometabolic risk factors (CMRF)	
Overweight or obesity	Body mass index ≥ 23 kg/m^2^ or Waist circumference ≥ 90 cm for male or ≥85 cm for female
Prediabetes or Diabetes mellitus	Fasting glucose ≥ 100 mg/dL or glycated hemoglobin A1c ≥ 5.7% or use of insulin or oral hypoglycemic agents
Elevated blood pressure	Blood pressure ≥ 130/85 mm Hg or use of anti-hypertensive medications
Hypertriglyceridemia	Triglycerides ≥ 150 mg/dL or use of lipid lowering drugs
Low high-density lipoprotein cholesterol (HDL)	HDL < 40 mg/dL for male or <50 mg/dL for female or use of lipid lowering drugs
SLD subtypes	(1) no SLD: HSI ≤ 31
	(2) MASLD: HSI > 31 + ≥1 of CMRFs + alcohol consumption < 20 g/day (female) < 30 g/day (male) (3) MetALD: HSI > 31 + ≥1 of CMRFs + alcohol consumption 20–50 g/day (female) 30–60 g/day (male) (4) ALD with MD: HSI > 31 + ≥1 of CMRFs + alcohol consumption > 50 g/day (female) > 60 g/day (male) (5) other SLDs: HSI > 31 and does not meet criteria for (1)–(4)

SLD, steatotic liver disease; MASLD, metabolic dysfunction-associated steatotic liver disease; MetALD, metabolic alcohol-associated liver disease; ALD with MD, alcoholic liver disease with metabolic dysfunction.

**Table 2:** Distribution of characteristics of the study sample (N = 20,141).

	Overall	SLD Categories				
		No SLD	MASLD	MetALD	ALD with MD	Other SLDs
	N = 20,141	N = 8383	N = 10,346	N = 947	N = 195	N = 270
Sex						
Male	8813 (43.8%)	3294 (39.3%)	4542 (43.9%)	743 (78.5%)	147 (75.4%)	87 (32.2%)
Female	11,328 (56.2%)	5089 (60.7%)	5804 (56.1%)	204 (21.5%)	48 (24.6%)	183 (67.8%)
Age						
Mean (SD)	50.8 (16.7)	48.4 (17.7)	53.6 (15.7)	47.9 (13.6)	42 (13.2)	38 (12.5)
Education level						
Middle school or below	6095 (30.3%)	2096 (25.0%)	3710 (35.9%)	238 (25.1%)	33 (16.9%)	18 (6.7%)
High school	6617 (32.9%)	2839 (33.9%)	3238 (31.3%)	346 (36.5%)	86 (44.1%)	108 (40.0%)
College or above	7429 (36.9%)	3448 (41.1%)	3398 (32.8%)	363 (38.3%)	76 (39.0%)	144 (53.3%)
Income level						
Lowest	3634 (18.0%)	1422 (17.0%)	2052 (19.8%)	121 (12.8%)	21 (10.8%)	18 (6.7%)
Low	4881 (24.2%)	1886 (22.5%)	2648 (25.6%)	240 (25.3%)	57 (29.2%)	50 (18.5%)
High	5601 (27.8%)	2359 (28.1%)	2827 (27.3%)	269 (28.4%)	60 (30.8%)	86 (31.9%)
Highest	6025 (29.9%)	2716 (32.4%)	2819 (27.2%)	317 (33.5%)	57 (29.2%)	116 (43.0%)
Smoking						
Yes	16,502 (81.9%)	6971 (83.2%)	8697 (84.1%)	518 (54.7%)	83 (42.6%)	233 (86.3%)
No	3639 (18.1%)	1412 (16.8%)	1649 (15.9%)	429 (45.3%)	112 (57.4%)	37 (13.7%)
Physical activity						
Yes	11,153 (55.4%)	4465 (53.3%)	5921 (57.2%)	536 (56.6%)	100 (51.3%)	131 (48.5%)
No	8988 (44.6%)	3918 (46.7%)	4425 (42.8%)	411 (43.4%)	95 (48.7%)	139 (51.5%)
HSI						
Median (Q1, Q3)	32.1 (28.9, 35.7)	28.4 (26.7, 29.7)	35.1 (32.9, 38.2)	35.1 (32.8, 38.5)	35.1 (32.6, 39.0)	32.0 (31.4, 33.1)
hs-CRP (g/L)						
Median (Q1, Q3)	0.5 (0.3, 1.0)	0.4 (0.3, 0.7)	0.7 (0.4, 1.3)	0.7 (0.4, 1.3)	0.7 (0.4, 1.4)	0.4 (0.3, 0.6)
AST (IU/L)						
Median (Q1, Q3)	20.0 (17.0, 25.0)	19.0 (16.0, 23.0)	21.0 (18.0, 26.0)	24.0 (19.0, 30.0)	24.0 (19.0, 31.0)	19.0 (16.0, 24.0)
ALT (IU/L)						
Median (Q1, Q3)	17.0 (13.0, 25.0)	13.0 (10.0, 17.0)	21.0 (16.0, 31.0)	25.0 (18.0, 36.0)	25.0 (19.0, 38.0)	21.0 (16.0, 28.0)

SD, standard deviation; SLD, steatotic liver disease; MASLD, metabolic dysfunction-associated steatotic liver disease; MetALD, metabolic alcohol-associated liver disease; ALD with MD, alcoholic liver disease with metabolic dysfunction; hs-CRP, high-sensitivity C-reactive protein; AST, aspartate aminotransferase; ALT, alanine aminotransferase.

**Table 3:** Log-linear regressions of the SLD types and hs-CRP levels among the overall sample.

	Univariate Model				Multivariate Model			
	*β*	SE	% Change (95% CI)	*p*	*β*	SE	% Change (95% CI)	*p*
SLD types								
No SLD	Ref.	Ref.	Ref.		Ref.	Ref.	Ref.	
MASLD	0.51	0.02	67.2 (61.8, 72.8)	<0.001	0.47	0.02	59.7 (54.5, 65.1)	<0.001
MetALD	0.56	0.04	75.1 (63.1, 87.9)	<0.001	0.47	0.04	60.6 (49.3, 72.8)	<0.001
ALD with MD	0.58	0.08	78.8 (54.2, 107.3)	<0.001	0.52	0.08	67.5 (44.4, 94.2)	<0.001
Other SLDs	−0.12	0.06	−11.5 (−21.3, −0.5)	0.041	−0.06	0.06	−5.8 (−16.2, 6.0)	0.323

SE, standard error; CI, confidence interval; SLD, steatotic liver disease; MASLD, metabolic dysfunction-associated steatotic liver disease; MetALD, metabolic alcohol-associated liver disease; ALD with MD, alcoholic liver disease with metabolic dysfunction; hs-CRP; high-sensitivity C-reactive protein.

**Table 4:** Sex-stratified analysis of the association between the SLD types and hs-CRP.

	Males				Females			
	*β*	SE	% Change (95% CI)	*p*	*β*	SE	% Change (95% CI)	*p*
SLD types								
No SLD	Ref.	Ref.	Ref.		Ref.	Ref.	Ref.	
MASLD	0.35	0.03	41.9 (35.1, 49.1)	<0.001	0.60	0.02	81.5 (73.6, 89.8)	<0.001
MetALD	0.38	0.04	46.8 (35.0, 59.6)	<0.001	0.61	0.08	84.3 (58.1, 114.8)	<0.001
ALD with MD	0.42	0.08	51.8 (30.0, 77.2)	<0.001	0.68	0.18	98.2 (38.0, 184.8)	<0.001
Other SLDs	−0.16	0.10	−14.9 (−29.8, 3.0)	0.097	0.01	0.08	1.0 (−13.0, 17.3)	0.893

SE, standard error; CI, confidence interval; SLD, steatotic liver disease; MASLD, metabolic dysfunction-associated steatotic liver disease; MetALD, metabolic alcohol-associated liver disease; ALD with MD, alcoholic liver disease with metabolic dysfunction; hs-CRP; high-sensitivity C-reactive protein.

## Data Availability

The raw data are accessible at https://knhanes.kdca.go.kr/knhanes (accessed on 23 August 2024).

## Supplementary Materials

The following supporting information can be downloaded at: https://www.mdpi.com/article/10.3390/biom14111468/s1, Table S1: Model with interaction terms between SLD type and sex (female) on hs-CRP values; Table S2: Log-linear regressions of the SLD types and hs-CRP levels among the overall sample population. Sensitivity analysis based on the alternative cutoff (HSI >36); Table S3: Sex-stratified analysis of the association between the SLD types and hs-CRP. Sensitivity analysis based on the alternative cutoff (HSI >36); Table S4: Log-linear regressions of the SLD types and AST and ALT among the overall sample (adjusted models); Table S5: Log-linear regressions of the SLD types and AST and ALT among the male sample (adjusted models); Table S6: Log-linear regressions of the SLD types and AST and ALT among the female sample (adjusted models); Table S7 Factors associated with having steatotic liver disease (HSI >31) on logistic regression model.
