# Supplementary material for: High-Sensitivity C-Reactive Protein Levels in Metabolic Dysfunction-Associated Steatotic Liver Disease (MASLD), Metabolic Alcohol-Associated Liver Disease (MetALD), and Alcoholic Liver Disease (ALD) with Metabolic Dysfunction
Source: Biomolecules. 2024 Nov 18;14(11):1468. doi: 10.3390/biom14111468 (PMC11591830; doi:10.3390/biom14111468)
Supplement: Supplementary file 1 [file biomolecules-14-01468-s001.zip › biomolecules-3290014-supplementary.pdf]

Table S1 Model with interaction terms between SLD type and sex (female) on hs-CRP values

	β	**SE**	**% change (95% CI)**	***P***
**SLD types**				
No SLD	Reference	Reference	Reference	
MASLD	0.11	0.05	11.7 (0.6, 24.1)	0.039
MetALD	0.18	0.11	19.4 (-4.5, 49.2)	0.119
ALD with MD	0.15	0.23	16.6 (-26.4, 84.6)	0.512
Other SLDs	-0.37	0.21	-30.9 (-54.1, 4.0)	0.077
**Interaction terms**				
MASLD × female	0.24	0.03	26.6 (18.8, 34.9)	< 0.001
MetALD × female	0.21	0.09	23.3 (4.0, 46.2)	0.016
ALD with MD × female	0.26	0.20	29.8 (-11.7, 90.9)	0.184
Other SLDs × female	0.19	0.12	21.3 (-4.7, 54.4)	0.117

SE, standard error; CI, confidence interval; SLD, steatotic liver disease; hs-CRP, high-sensitivity C-reactive protein; MASLD, metabolic dysfunction-associated steatotic liver disease; MetALD, metabolic alcohol-associated liver disease; ALD with MD, alcoholic liver disease with metabolic dysfunction

Table S2 Log-linear regressions of the SLD types and hs-CRP levels among the overall sample population. Sensitivity analysis based on the alternative cutoff (HSI >36)

	**Univariate model**				**Multivariate model**			
	β	**SE**	**% change (95% CI)**	***P***	***β***	**SE**	**% change (95% CI)**	***P***
**SLD types**								
No SLD	Reference	Reference	Reference		Reference	Reference	Reference	
MASLD	0.60	0.02	82.6 (75.7, 89.8)	< 0.001	0.57	0.02	77.3 (70.5, 84.2)	< 0.001
MetALD	0.59	0.05	81.2 (63.4, 100.9)	< 0.001	0.53	0.06	70.4 (52.9, 89.9)	< 0.001
ALD with MD	0.67	0.11	96.1 (59.0, 142.0)	< 0.001	0.63	0.11	88.6 (52.6, 133.0)	< 0.001
Other SLDs	-0.01	0.29	-0.7 (-43.7, 75.2)	0.981	0.10	0.29	10.8 (-36.9, 94.7)	0.720

SE, standard error; CI, confidence interval; SLD, steatotic liver disease; hs-CRP, high-sensitivity C-reactive protein; MASLD, metabolic dysfunction-associated steatotic liver disease; MetALD, metabolic alcohol-associated liver disease; ALD with MD, alcoholic liver disease with metabolic dysfunction

Table S3 Sex-stratified analysis of the association between the SLD types and hs-CRP. Sensitivity analysis based on the alternative cutoff (HSI >36)

	**Males**				**Females**			
	β	**SE**	**% change (95% CI)**	***P***	***β***	**SE**	**% change (95% CI)**	***P***
**SLD types**								
No SLD	Reference	Reference	Reference		Reference	Reference	Reference	
MASLD	0.48	0.03	61.9 (53.6, 70.6)	< 0.001	0.69	0.03	98.8 (88.4, 109.8)	< 0.001
MetALD	0.45	0.06	56.3 (39.2, 75.5)	< 0.001	0.85	0.12	134.5 (86.7, 194.6)	< 0.001
ALD with MD	0.47	0.11	60.4 (28.5, 100.3)	< 0.001	1.42	0.24	314.4 (159.0, 562.8)	< 0.001
Other SLDs	0.14	0.17	15.3 (-18.1, 62.3)	0.415	0.00	0.80	0.2 (-79.1, 380.6)	0.998

SE, standard error; CI, confidence interval; SLD, steatotic liver disease; hs-CRP, high-sensitivity C-reactive protein; MASLD, metabolic dysfunction-associated steatotic liver disease; MetALD, metabolic alcohol-associated liver disease; ALD with MD, alcoholic liver disease with metabolic dysfunction

Table S4. Log-linear regressions of the SLD types and AST and ALT among the overall sample (adjusted models)

	AST				ALT			
	β	**SE**	**% change (95% CI)**	***P***	***β***	**SE**	**% change (95% CI)**	***P***
**SLD types**								
No SLD	Ref.	Ref.	Ref.		Ref.	Ref.	Ref.	
MASLD	0.11	0.01	11.2 (10.1, 12.4)	< 0.001	0.54	0.01	71.2 (68.6, 73.8)	< 0.001
MetALD	0.20	0.01	21.9 (18.8, 25.1)	< 0.001	0.56	0.02	75.9 (69.4, 82.6)	< 0.001
ALD with MD	0.23	0.03	25.5 (19.2, 32.2)	< 0.001	0.59	0.04	79.8 (66.8, 93.9)	< 0.001
Other SLDs	0.07	0.03	7.3 (2.0, 12.8)	0.006	0.58	0.03	79.0 (68.1, 90.7)	< 0.001

SE, standard error; CI, confidence interval; SLD, steatotic liver disease; MASLD, metabolic dysfunction-associated steatotic liver disease; MetALD, metabolic alcohol-associated liver disease; ALD with MD, alcoholic liver disease with metabolic dysfunction; AST, aspartate aminotransferase; ALT, alanine aminotransferase

Table S5. Log-linear regressions of the SLD types and AST and ALT among the male sample (adjusted models)

	AST				ALT			
	β	**SE**	**% change (95% CI)**	***P***	***β***	**SE**	**% change (95% CI)**	***P***
**SLD types**								
No SLD	Ref.	Ref.	Ref.		Ref.	Ref.	Ref.	
MASLD	0.13	0.01	13.8 (11.9, 15.7)	< 0.001	0.61	0.01	84.7 (80.4, 89.0)	< 0.001
MetALD	0.23	0.02	26.0 (22.3, 29.9)	< 0.001	0.63	0.02	88.5 (80.8, 96.6)	< 0.001
ALD with MD	0.25	0.03	28.9 (21.5, 36.8)	< 0.001	0.64	0.04	91.3 (76.1, 107.9)	< 0.001
Other SLDs	0.05	0.04	5.2 (-2.4, 13.4)	0.186	0.59	0.05	80.4 (64.4, 98.0)	< 0.001

SE, standard error; CI, confidence interval; SLD, steatotic liver disease; MASLD, metabolic dysfunction-associated steatotic liver disease; MetALD, metabolic alcohol-associated liver disease; ALD with MD, alcoholic liver disease with metabolic dysfunction; AST, aspartate aminotransferase; ALT, alanine aminotransferase

Table S6. Log-linear regressions of the SLD types and AST and ALT among the female sample (adjusted models)

	AST				ALT			
	β	**SE**	**% change (95% CI)**	***P***	***β***	**SE**	**% change (95% CI)**	***P***
**SLD types**								
No SLD	Ref.	Ref.	Ref.		Ref.	Ref.	Ref.	
MASLD	0.07	0.01	6.9 (5.5, 8.4)	< 0.001	0.42	0.01	52.9 (49.9, 56.0)	< 0.001
MetALD	0.11	0.03	11.6 (6.2, 17.2)	< 0.001	0.43	0.04	53.0 (42.5, 64.4)	< 0.001
ALD with MD	0.15	0.04	16.5 (7.2, 26.6)	< 0.001	0.46	0.07	58.1 (37.4, 81.9)	< 0.001
Other SLDs	0.08	0.03	7.8 (1.0, 15.1)	0.023	0.56	0.04	75.0 (61.4, 89.9)	< 0.001

SE, standard error; CI, confidence interval; SLD, steatotic liver disease; MASLD, metabolic dysfunction-associated steatotic liver disease; MetALD, metabolic alcohol-associated liver disease; ALD with MD, alcoholic liver disease with metabolic dysfunction; AST, aspartate aminotransferase; ALT, alanine aminotransferase

Table S7 Factors associated with having steatotic liver disease (HSI >31) on logistic regression model

	**Odds Ratio (95% confidence interval)**
Sex	
Male	Reference
Female	0.59 (0.54, 0.64)
Age	
Continuous scale	1.02 (1.01, 1.02)
Education level	
Middle school or below	Reference
High school	0.80 (0.72, 0.89)
College or above	0.79 (0.70, 0.88)
Income level	
Lowest	Reference
Low	1.20 (1.07, 1.34)
High	1.20 (1.07, 1.35)
Highest	1.10 (0.98, 1.24)
Smoking	
No	Reference
Yes	1.05 (0.95, 1.15)
Physical activity	
No	Reference
Yes	0.97 (0.91, 1.04)

HSI, hepatic steatosis index
